# A multivariate analysis for delayed healing of facial muscle spasm after microvascular decompression

**DOI:** 10.12669/pjms.343.15015

**Published:** 2018

**Authors:** Tianyu Lu, Yifan Xu, Wu Xu, Yuxiang Dai, Weibang Liang, Wei Jin

**Affiliations:** 1 Tianyu Lu Department of Neurosurgery, Nanjing Drum Tower Hospital, Clinical College of Nanjing Medical University, Nanjing 210008, P. R. China; 2 Yifan Xu Department of Neurosurgery, Nanjing Drum Tower Hospital, Clinical College of Nanjing Medical University, Nanjing 210008, P. R. China; 3 Wu Xu Department of Neurosurgery, Nanjing Drum Tower Hospital, Clinical College of Nanjing Medical University, Nanjing 210008, P. R. China; 4 Yuxiang Dai Department of Neurosurgery, Nanjing Drum Tower Hospital, Clinical College of Nanjing Medical University, Nanjing 210008, P. R. China; 5 Weibang Liang Department of Neurosurgery, Nanjing Drum Tower Hospital, Clinical College of Nanjing Medical University, Nanjing 210008, P. R. China; 6 Wei Jin Department of Neurosurgery, Nanjing Drum Tower Hospital, Clinical College of Nanjing Medical University, Nanjing 210008, P. R. China

**Keywords:** Facial muscle spasm, Microvascular decompression, Delayed healing

## Abstract

**Objectives:**

To analyze the related factors for delayed healing of facial muscle spasm after microvascular decompression.

**Methods:**

After microvascular decompression, 116 of 425 patients with delayed healing were followed up, and their clinical data were analyzed.

**Results:**

The incidence rate of postoperative delayed healing was 27.3%, which was not correlated with gender, age or intraoperative vascular compression. However, it was correlated with disease course, severity of preoperative symptoms, arteriosclerosis and abnormal facial muscle response. The duration of delayed healing was positively correlated with preoperative disease course.

**Conclusions:**

Delayed healing is a common phenomenon after microvascular decompression for facial muscle spasm, with an elusive reason. Therefore, the treatment outcomes should be evaluated after one year of follow-up.

## INTRODUCTION

Facial Muscle Spasm (FMS) refers to the stiffness or clonus of muscles controlled by homolateral facial nerves, which is painless, intermittent, unspontaneous and irregular. First starting from the orbicularis oculi muscle in most cases, FMS induces twitching downwards gradually, involving the orbicularis oris muscle and facial expression muscle. Upon tension and rage, clonus is aggravated, with its number and frequency evidently decreasing in the case of calmness and sleeping. FMS mostly occurs on the same side of face,^1^ and commonly endangers people aged over 40 years old. The morbidity rate is approximately 11/1 million.^2^ Patients suffer both physically and psychologically, even accompanied by severely affected social activities. The currently available therapies for FMS, such as drugs and radiofrequency therapy, usually do not work or have many complications and easy recurrence.^3^ Nowadays, FMS has been widely attributed to vascular compression, and microscopic techniques for neurosurgeries have been burgeoning. Therefore, Microvascular Decompression (MVD) is given first priority in FMS treatment.^4^,^5^

In 1875, Schultze first described FMS, but did not further study the pathogenesis. FMS can be classified into primary and secondary types. The pathogenesis of secondary FMS can be clarified by using neurological examination methods and auxiliary strategies, including tumors in the cerebellopontine angle, inflammation, arterial aneurysm, brainstem encephalitis, syringobulbia, craniocerebral injury and symptoms of later-stage facial nerve paralysis.^6^ Besides, it is complicated with other symptoms of cranial nerve damage for identification. However, the incidence rate of secondary FMS is rather low, with most cases being primary.

FMS has been ascribed to vascular compression,^7^ i.e. blood vessels compress the Root Exit Zone (REZ) of facial nerve to damage the myelin sheath. As a result, nerve fibers underwent ectopic impulses through cross-synaptic transmission, leading to FMS finally. By establishing an animal model of the stylomastoid foramen exit with demyelination induced by chronic vascular compression, Chon et al.^8^ verified that delayed response of the facial motor nucleus mainly contributed to FMS. In other words, chronic vascular compression produced antidromic impulse to stimulate the motor nucleus and opened dormant synapses controlling different parts of facial nerve, resulting in Abnormal Muscle Response (AMR) and synkinesis. Consequently, the excitability of the facial motor nucleus was enhanced, and facial muscles unspontaneously twitched.

MVD exerts therapeutic effects by pushing offending vessels away from facial nerve roots with a cotton piece. In the past decades, MVD has been widely applied in clinical practice owing to safety and efficacy. It is now well-established that vascular loops play key roles in inducing FMS through the pulsatile compression of REZ. The offending vessels leading to compression are mostly elongated, expanded, hardened, tortuous vertebrobasilar arteries. In some cases, facial nerve roots even have impressions, being concomitant with local attenuation and color changes.^9^

In this study, we followed up 106 of 415 patients with delayed healing after MVD, and analyzed their clinical data, aiming to determine the related factors.

## METHODS

### Clinical Data

A total of 425 FMS patients were selected, including 191 males and 234 females aged from 17 to 75 years old, 49.5 on average. The disease courses ranged from 2 months to 20 years, 4.2 years on average. This study has been approved by the ethics committee of our hospital, and written consent has been obtained from all patients. Before hospitalization, all patients had received conservative therapies such as acupuncture and moxibustion, blocking therapy, injection of Botulinum toxin and administration of carbamazepine. After hospitalization, they all received cranial CT (scan of the posterior cranial fossa) and/or MRI examination to exclude intracranial space-occupying lesion and other pathogenic factors.

### Surgical Methods

General anesthesia was conducted by endotracheal intubation in combination with intravenous inhalation. In the supine position of the unaffected side, the head was moved downwards by 15° and rotated towards such side by 10°.

With the neck slightly flexing forwards, mastoid process of the affected side was located basically parallel to the operating table in the highest position, which helped to maintain the same direction of the microscope optical axis and surgical approach. A 3~5 cm vertical incision was made parallel to and within 0.5 cm away from the postauricular hair line. The bone window had a diameter of 1.5~2.0 cm, with the frontal boundary and the lower boundary approaching the sigmoid sinus and the skull base respectively. The dura mater was cut open in a “⊥” shape. Under the microscope, glossopharyngeal and vagus nerves were exposed, the arachnoid membrane at nerve roots was scissored open, and flocculi were pulled away. Meanwhile, the head position and microscope optical axis were adjusted, exposing REZ of facial nerve. After offending vessels were identified, they were dissociated and pushed away from REZ. Then a piece of Teflon cotton with appropriate size was put between the vessels and the brainstem. Subsequently, the skull was closed routinely. During surgery, real-time brainstem auditory evoked potential monitoring and AMR monitoring were carried out.

### Statistical Analysis

All data were analyzed by SAS 9.1. Inter-group comparisons were performed by the χ^2^ test, Fisher’s exact test and Wilcoxon rank sum test. The factors affecting the duration of delayed healing were studied with multivariate linear regression analysis. P<0.05 was considered statistically significant.

## RESULTS

Among the 425 patients, the FMS symptoms immediately disappeared after surgery in 267 cases (62.8%), which was set as the immediate healing group. 116 cases (27.3%) still had various degrees of facial convulsion or recurrence after the symptoms disappeared, which was set as the delayed healing group. In the 116 patients, most of the FMS symptoms were alleviated during recurrence compared with before surgery, and completely disappeared 7 days to 8 months after surgery, 6 weeks on average. All patients were followed up for 1 to 4 years (2.3 years on average); the symptoms disappeared completely in 364 cases (85.6%); the symptoms were significantly alleviated but not completely disappeared in 43 cases (10.2%); there were 9 ineffective cases (2.1%), 9 recurrent cases (2.1%), with the total effective rate of 95.8%.

Multivariate analysis was used to analyze whether the variables such as gender, age, history, severity of preoperative symptoms and diameter of the offending arteries (bulky arteries means the vertebral arteries, the posterior inferior cerebellar arterial trunk, and the anterior inferior cerebellar arterial trunk; arterioles include the posterior inferior cerebellar arterial branch and the anterior inferior cerebellar arterial branch), atherosclerosis and intraoperative monitoring of AMR results were related to delayed healing.

There was no significant difference in gender, age and offending arteries between the two groups (both P>0.05). In other words, the gender, age and diameter of the offending arteries of the patients were not correlated with the occurrence of postoperative delayed healing. The two groups had significant differences in history, severity of symptoms, arteriosclerosis and intraoperative AMR monitoring (all P<0.05), i.e. the patients who had a long history, severe symptoms, arteriosclerosis and whose AMR did not completely disappear during intraoperative monitoring were prone to delayed healing after surgery (Table-I).

**Table-I:** Factors affecting healing.

Factor	Immediate healing	Delayed healing	Statistical data	P
Gender (n)			χ^2^=0.52	0.479
Male	116	47		
Female	151	69		
Age (year)	48.5	50	t=1.07	0.286
Disease history (year)	3.8	5.9	t=5.55	<0.001
Degree of symptoms Φ			Fisher	0.000
Mild	8	4		
Mild-moderate	57	2		
Moderate	67	20		
Moderate-severe	85	53		
Severe				
Offending artery (n)			χ^2^=1.94	0.143
Thin	159	60		
Thick	108	56		
AMR monitoring (n) Φ			Fisher	0.000
Disappear	151	36		
Partly disappear	7	17		
Unchanged	4	8		
Arteriosclerosis			χ^2^=5.48	0.015
Yes	253	98		
No	14	18		

Φ Fisher’s exact test was used for inter-group comparisons.

In the influencing factors of delayed healing duration, the duration of delayed healing was taken as the dependent variable, and the age (whether the patients were aged greater than 50 years old), gender, history, severity of symptoms, atherosclerosis, intraoperative AMR and the diameter of offending arteries were used as independent variables to screen the variables using the stepwise regression method. Finally, the independent variables of history, gender and severity of symptoms entered into the model, and the screened independent variables had no multicollinearity, i.e. no clear correlation.

There was a linear regression relationship between history, gender, severity of symptoms and duration of delayed healing. According to the regression coefficient, it can be concluded that gender is negatively correlated with the duration of delayed healing, that is, male patients have a longer duration of delayed healing than that of female ones. The history and severity of symptoms are positively correlated with the duration of delayed healing, that is, the longer the history, the more severe the symptoms, the longer it will take before healing. The regression coefficient of the history was the largest, indicating that the history has the greatest effect on the duration of delayed healing (Table-II).

**Table-II:** Factors affecting delayed healing.

Factor	N	Duration of delayed healing	Regression coefficient	Statistical data	P
Gender (n)			-0.19	-2.57	<0.01
Male	47	46.5			
Female	69	50			
Disease history (year)			0.81	11.5	<0.01
≤10	104	38			
>10	12	196			
Degree of symptoms			0.18	2.67	<0.01
Mild, mild-moderate	6	11			
Moderate	20	23			
Moderate-severe, severe	90	55.5			

## DISCUSSION

The facial nerve exits the brain stem from the pontobulbar sulcus on the upper edge of the upper olive fossa, of which the central segment clings to the pontine surface, leaves the brain stem after 8-10 mm away outwards, and then migrates to the cisternal segment of the facial nerve through the Obsterteiner-Redlich Transition Zone (TZ) of about 2mm. In the facial nerve root, the myelin sheath of the central segment is composed of oligodendrocytes, and that of the peripheral segment Schwann cells. The conical terminal of the central myelin sheath is surrounded by peripheral myelin sheath to form TZ.^10^ It has been considered that pulsating and crossing vascular compression of TZ are the main causes of FMS and are conceptually equivalent to facial nerve REZ.^11^ But later studies have shown that only a small number of patients with FMS have VZ vascular compression, of which the vast majority are found in the central segment. Since the segment is more sensitive to vascular compression, the outer circumference wrapped by Schwann cells is more tolerant to vascular compression, so the offending vessels are mostly found in the central segment. In order to better describe the specific position of the vascular nerve root, Tomii et al.^12^ refined the concept of REZ, and described the REZ of facial nerve as the REZ point (REx P) and the separated site of medial facial nerve and pontine surface as the nerve dissociation point (RDP). The facial nerves between REx P and RDP are close to the pontine surface, and the middle consists of close-knit pia mater and connective tissue. In short, the scope of facial nerve REZ should include REx P, AS, RDP and TZ, i.e. within the range of about 10mm away from the point of facial nerve REZ. Yamakami et al.^13^ established an animal model through giving chronic electrical stimulation in the facial nerve near the central segment every day and giving electrical stimulation in the facial nerve temporal branch after a period of time, to record the AMR waveform in the buccinator, which inferred that the pathogenesis of FMS was the physical stimulation of offending vascular compression, leading to increased excitability of facial nuclei.

After decades of development and improvement, MVD has become the preferred therapy of FMS owing to its features of high cure rate and good security, in particular, completely retaining the characteristics of blood vessels and nerve function. The total effective rate of treatment was 87.5%-99.3%. However, the symptoms of many patients do not disappear immediately after surgery, but gradually disappear after a period of time, known as delayed healing. Oh et al.^14^ reported the delayed healing of FMS after MVD, finding that the postoperative symptoms of about 13% to 60% of patients did not immediately disappear, but gradually disappeared after 1 week to six months or even more than one year. Among the 425 patients in this study, the FMS symptoms of 267 cases (62.8%) immediately disappear after surgery, and in the remaining patients with varying degrees of postoperative facial convulsions, the symptoms of 116 cases (27.3%) completely disappeared 7 days to 8 months after surgery, 6 weeks on average, with a total effective rate of 95.8%.

As for the reasons of delayed healing, it has been reported that^15^ the immediate healing of FMS patients after MVD is due to the fundamental cause of the direct pulsating impact of compression of offending vessels on the REZ of facial nerve, so the symptoms disappear immediately after vascular decompression. And for some patients with longer disease course and/or severe vascular compression (including offending vessels with vertebral artery, posterior inferior cerebellar arterial trunk or anterior inferior cerebellar arterial trunk and other bulky blood vessels), the offending vessels compress the REZ of facial nerve, and cause local severe demyelinating lesions and/or strong over-excitement of facial nerve movement nucleus in the zone. Although the vascular compression factors are released after MVD, it will need a period of time to complete the regenerative repair of facial nerve root demyelinating lesion and/or enable the excessive excitability of motor nucleus of facial nerve to return to normal, resulting in delayed healing. In the statistical analysis of the patients in this study, it was found that the occurrence of delayed healing was independent of the gender, age and the diameter of compressed arteries, but was related to the length of preoperative history, severity of symptoms and arteriosclerosis. Nevertheless, the above results cannot be used to explain the occurrence of delayed healing, and its internal mechanism needs to be further clarified.

We also found that the patients with a long history, more severe symptoms and the intra-operative occurrence of arteriosclerosis may also experience a long duration in case of delayed healing. This finding will help to observe the surgical efficacy and make correct judgments, and generally presume the duration of delayed healing according to the length of preoperative history. Moreover, 268 patients in this study underwent AMR monitoring during surgery, and were found that the waveform changes were of a certain significance to the prediction of postoperative delayed healing, which was basically consistent with the findings of Joo et al.^16^ We found that the complete disappearance of abnormal waves often indicated good immediate effects, and no change in abnormal waves or volatility decline but no complete disappearance might prompt the possibility of the occurrence of postoperative delayed healing. In this case, we need to patiently explore whether there is any missing of offending vessels or thorough decompression in the REZ of facial nerve. As long as the surgery is identified with a clear vascular compression and satisfactory decompression, we can just make patient follow-up observation rather than worry about the efficacy. In view of the existence of delayed healing, scholars suggest that the identification of efficacy on FMS patients be made only after at least 6 months of follow-up after MVD. In the process of long-term follow-up for 425 patients, we found that two cases appeared to have delayed healing 6 months after surgery, and one case had its symptoms completely disappeared 8 months after surgery, which was the longest. Therefore, it is necessary to conduct evaluation of the efficacy after more than one year of continuous follow-up.

In summary, during MVD, the correct judgments and accurate decompression operation on offending vessels can improve the overall effect of surgery, but will not significantly reduce the proportion of delayed healing. We do not recommend conducting the second surgical exploration immediately when the surgery is determined ineffective once FMS symptoms are not alleviated 3 days postoperatively. For MVD physicians with extensive surgical experience, there is no need for the second surgery in patients with poor efficacy immediately after surgery in a short term. Further basic and clinical studies on the postoperative delayed healing of FMS after MVD will help us understand the nature of FMS disease at a more in-depth level so as to improve its surgical outcomes.

### Authors’ Contributions

**WL and WJ:** Designed this study and significantly contributed to manuscript preparation.

**TL, YX, WX & YD:** Performed this study and drafted this manuscript.
